# The effect of community health worker–led education on women’s health and treatment–seeking: A cluster randomised trial and nested process evaluation in Gujarat, India

**DOI:** 10.7189/jogh.07.020404

**Published:** 2017-12

**Authors:** Sapna Desai, Ajay Mahal, Tara Sinha, Joanna Schellenberg, Simon Cousens

**Affiliations:** 1 Faculty of Epidemiology and Population Health, London School of Hygiene and Tropical Medicine, London, UK; 2 Self Employed Women’s Association, Ahmedabad, Gujarat, India; 3 Nossal Institute for Global Health, University of Melbourne, Melbourne, Australia

## Abstract

**Background:**

A community–based health insurance scheme operated by the Self–Employed Women’s Association in Gujarat, India reported that the leading reasons for inpatient hospitalisation claims by its members were diarrhoea, fever and hysterectomy – the latter at the average age of 37. This claims pattern raised concern regarding potentially unnecessary hospitalisation amongst low–income women.

**Methods:**

A cluster randomised trial and mixed methods process evaluation were designed to evaluate whether and how a community health worker–led education intervention amongst insured and uninsured adult women could reduce insurance claims, as well as hospitalisation and morbidity, related to diarrhoea, fever and hysterectomy. The 18–month intervention consisted of health workers providing preventive care information to women in a group setting in 14 randomly selected clusters, while health workers continued with regular activities in 14 comparison clusters. Claims data were collected from an administrative database, and four household surveys were conducted amongst a cohort of 1934 randomly selected adult women.

**Results:**

30% of insured women and 18% of uninsured women reported attending sessions. There was no evidence of an intervention effect on the primary outcome, insurance claims (risk ratio (RR) = 1.03; 95% confidence interval (CI) 0.81, 1.30) or secondary outcomes amongst insured and uninsured women, hospitalisation (RR = 1.05; 95% CI 0.58, 1.90) and morbidity (RR = 1.09; 95% CI 0.87, 1.38) related to the three conditions. The process evaluation suggested that participants retained knowledge from the sessions, but barriers to behaviour change were not overcome.

**Conclusions:**

We detected no evidence of an effect of this health worker–led intervention to decrease claims, hospitalisation and morbidity related to diarrhoea, fever and hysterectomy. Strategies that capitalise on health workers’ role in the community and knowledge, as well as those that address the social determinants of diarrhoea, fever and the frequency of hysterectomy – such as water and sanitation infrastructure and access to primary gynaecological care – emerged as areas to strengthen future interventions.

Since the Alma–Ata declaration, community health workers (CHWs), also known as lay health workers, have been promoted as a key component of primary health care strategies aimed at women and children [1]. CHWs have also been shown to be uniquely positioned to influence behavior change, through their use of indigenous knowledge and ability to communicate with empathy and locally appropriate language [2]. Evidence synthesised through meta–analyses, qualitative syntheses and disease–focused reviews thus far suggests that CHWs have the potential to improve knowledge, behaviour and health outcomes [3–8]. A 2010 Cochrane systematic review and meta–analysis of interventions involving lay health workers indicated moderate evidence of their potential to improve immunisation coverage, breastfeeding and adherence to tuberculosis treatment, primarily through one–to–one visits and linking women to health systems [4]. Evidence, albeit limited, also suggests that CHW–led education delivered to women in a group setting can improve knowledge and preventive behavior [9–16]. This paper reports on the findings of a cluster randomised trial and nested process evaluation of a CHW–led group health education intervention to improve women’s health and treatment–seeking behaviour in a low–income setting in Gujarat, India.

## Study setting

Gujarat, though one of India’s wealthier states, performs close to national averages with regards to many health indicators. The last (2015–6) National Family Health Survey reported an infant mortality rate of 34/1000 live births and that only one–half (50.4%) of children between 12–23 months were fully immunised [17]. Utilisation of health services in Gujarat, as in most of India, is largely financed by individual households. Outpatient and inpatient care are predominantly sought in the private sector [18]. In 2009, Gujarat initiated roll–out of Rashtriya Swasthya Bima Yojana (RSBY), a government–financed health insurance scheme that provides hospitalisation coverage up to Rs 30 000 (US$ 442, 12.19.2016) for families identified to be below the poverty line [19]. In 2011, Gujarat recruited close to 30 000 village health workers (known as Accredited Social Health Activists, or ASHAs), one per 1000 population to cover its 18 539 villages [20].

The intervention was designed with the Self–Employed Women’s Association (SEWA), a trade union of over 1.5 million women workers in India’s informal economy, whose members typically have insecure employment and limited access to social protection. SEWA operates a community health worker–led health program and insurance scheme for its members, VimoSEWA, that provides up to Rs.5000 (74 USD, 19 December 2016) coverage for inpatient hospitalisation that exceeds 24 hours in exchange for annual premium payments by members.

## Intervention

A 2009 analysis of 12 027 VimoSEWA hospitalisation claims reported that two of the leading reasons for inpatient hospitalisation amongst adult women were diarrhea and fever, the latter considered primarily related to malaria [21]. The third leading reason for insurance claims was hysterectomy, at a relatively low average age of 37. VimoSEWA was surprised by the high proportion of hospitalisation for diarrhoea and fever – seemingly common, preventable ailments. The frequency and age at hysterectomy suggested that some procedures may not have been medically indicated and were thus avoidable. Given that diarrhoea, fever and hysterectomy comprised over 40% of VimoSEWA’s claims, SEWA aimed to design a scalable intervention to reduce claims, hospitalisation and morbidity related to the three conditions. If effective, the intervention would protect members from unnecessary hospitalisation as well as improve VimoSEWA’s financial sustainability.

The aim of the intervention was to (i) raise awareness on prevention and immediate treatment for malaria–related fever and diarrhea and (ii) improve knowledge of hysterectomy and its side effects, in order to reduce medically unnecessary procedures. The intervention focused on group health education sessions implemented by its CHW team; this approach was viable with respect to the financial and human resources available. Operationally, SEWA defined health education as a tool to improve knowledge and change women’s attitudes and behavior through information, dissemination and discussion. Further, since SEWA’s CHWs were seasoned local leaders and activists, group education sessions could potentially engage women in community action. At the time of the intervention, SEWA CHWs conducted limited group health education programs, none of which addressed diarrhoea, fever/malaria or hysterectomy. Both intervention and control areas were exposed to information through government health programs, including ASHA home visits to mothers and children and limited media messaging. Messages included information on malaria and diarrhoea and did not address gynaecological ailments. However, since ASHAs were neither trained nor incentivised to conduct health education, SEWA felt a group–based intervention could fill an important gap in existing services. SEWA CHWs in intervention areas implemented three to five group health education sessions monthly with adult women over an 18–month period, while comparison area CHWs continued with regular activities (**Table 1**).

**Table 1:** CHW activities in comparison and intervention areas

Activity	Intervention	Comparison
Home visits and group education on common illnesses (excluding diarrhoea, malaria and hysterectomy)	×	×
Accompanied referral to health services	×	×
Medicine sales and insurance promotion	×	×
Linkages with government providers	×	×
Activate Village Health and Sanitation Committees	×	×
Group education sessions on hysterectomy with film viewings	×	
Communication tools/handouts on hysterectomy	×	
Group education on diarrhoea with ORS demonstrations	×	
Group education on fever/malaria with interactive games	×	
Wall paintings on diarrhoea and malaria	×	
Education sessions on sanitation linkages and programs	×	
Monthly refresher training for CHWs	×	

ORS – oral rehydration salts, CHW – community health worker

## METHODS

As the intervention was implemented at the CHW level, a cluster randomised trial was designed to evaluate the effect of the intervention on three outcomes: claims rates (primary outcome), hospitalisation and morbidity related to diarrhoea, fever and hysterectomy. Clusters were defined as the discrete geographical areas served by one CHW. The number of clusters included in the trial, 28 in total, was determined by the number of CHWs available in areas where VimoSEWA operates. The intervention was implemented in 14 randomly–selected CHW catchment areas of Ahmedabad city and rural areas of Ahmedabad district, with 14 comparison clusters. Randomisation was stratified by urban and rural location, as urban rates of claim submission had been observed to be higher than in rural areas in two previous studies at VimoSEWA [21,22]. Treatment allocation was assigned through randomly generated numbers and announced in a CHW meeting. Data collectors were not informed of cluster allocations.

### Claims

Reduction in claims submission was measured by utilising all VimoSEWA insurance claims submitted in the intervention and control areas over the intervention period, as recorded in VimoSEWA’s claims database. VimoSEWA considered the minimum worthwhile intervention effect to be a 30–40% reduction in claims for the three conditions. The cooperative had moved towards a sustainable model without external funding support; a smaller reduction in claims would not have justified funding a health intervention from its operational costs. The between–cluster coefficient of variation (*k*) was estimated to be 0.28 using data on claims submission rates in 2008–2009 [23]. The study was estimated to have 77% power (*P* < 0.05, 2–sided test) to detect a 40% reduction in insurance claims for diarrhea, fever and hysterectomy.

### Hospitalisation and morbidity

**Figure 1:**
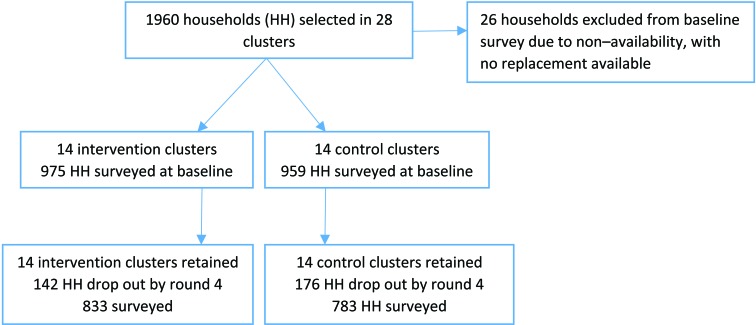
Cluster and survey participation.

Data on hospitalisation and morbidity rates related to the three conditions were collected through household surveys. Both insured and uninsured women were included in the household survey to enable the investigation of predictors of insurance coverage and to investigate whether the effect of the intervention varied with insurance status. A sample size of 35 uninsured and 35 insured households per cluster was chosen – a total of 1960 households across 28 clusters. Household listings of insured women were provided by VimoSEWA. A listing of uninsured households was compiled by following CHWs on daily rounds. Households were randomly selected through computer generated numbering. A baseline survey was conducted from January to March 2010, followed by three survey rounds at six–month intervals following implementation of the intervention. An adult woman was selected for interview in each household: the same primary VimoSEWA policy holder or SEWA member in uninsured households was interviewed at each round. A total of 980 uninsured and 954 uninsured adult women were surveyed at baseline. Survey data were double–entered into a Microsoft Access database. A supervisor observed a random sub–set of interviews and checked each survey form manually before data entry. Attrition increased at each round, primarily due to demolition of slum pockets in Ahmedabad city and rural pre–monsoon seasonal migration: a total of 1616 households were surveyed in the final round (**Figure 1**).

### Statistical methods

**Figure 2:**
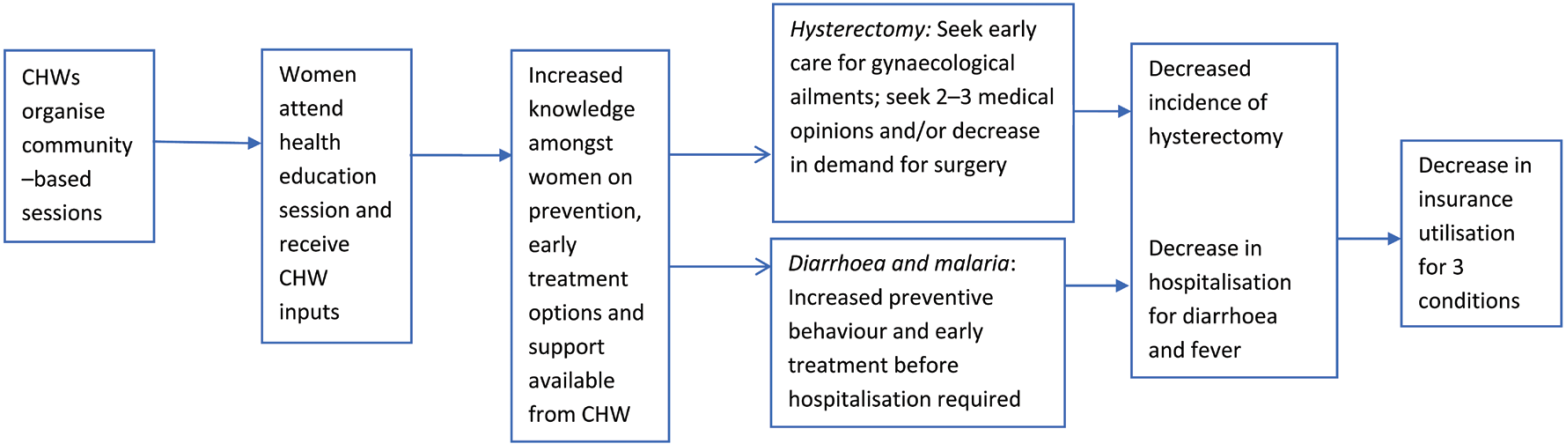
Intervention casual chain.

Analysis was by intention to treat. In the initial analysis, women’s insurance status at baseline was used to define the insured and uninsured groups. A Poisson regression model with cluster–level random effects to account for between–cluster variation was fitted to estimate the effect of the intervention on claims rates for the three conditions [24]. Effect estimates were adjusted for rural–urban location and cluster–level baseline claims rates. Likelihood ratio tests comparing models with and without the intervention effect were performed to obtain p values. Analyses of the effect of the intervention on hospitalisation and morbidity rates for the three conditions were conducted using similar methods, adjusting for survey round, insurance status, rural/urban location and cluster–level baseline rates. Effect modification by rural/urban location was examined for all three outcomes and by insurance status for hospitalisation and morbidity. Lastly, a process evaluation collected quantitative and qualitative data at each step in the hypothesised causal chain (**Figure 2**).

### Ethics and consent

Representatives of the clusters, drawn from SEWA’s membership–based health cooperative, provided approval prior to randomisation. A board constituted by SEWA’s Health Cooperative Executive Committee and the Ethics Committee of the London School of Hygiene and Tropical Medicine granted ethical approval for the intervention, evaluation and qualitative research. In light of low literacy levels in the study area, all households provided oral informed consent to participate in the survey, as approved by the local ethics board. The study was registered as ISRCTN21290274. Reporting follows the CONSORT guidelines and extension for cluster randomised trials.

## RESULTS

### Baseline comparability

Based on the demographic characteristics recorded in VimoSEWA’s administrative databases, intervention and control arms were generally balanced, with the exception of differences in the proportions of agricultural and home–based workers (**Table 2**). Claims rates based on individual–level data and cluster summaries were similar (5.4 and 5.3 per 100 person–years). The between cluster coefficients of variation (*k*) in claims rates, estimated using baseline data, were 0.46 (urban) and 0.66 (rural).

**Table 2:** Overview of baseline demographic characteristics, VimoSEWA membership database

	Intervention (n = 1839)	Comparison (n = 1719)
**Demographic variables:**		
Mean age	37.7	37.1
% married	83.8	85.3
% widowed	10.1	9.4
**Occupation:**		
% agricultural	34.8	44.7
% service	37.3	36.5
% home–based	17.7	10.7
% unemployed	10.1	8.0
**Baseline claims rate** (/100 person–years)	5.7	5.0

Similarly, household survey data indicated that baseline demographic characteristics were largely balanced across intervention and comparison arms, including baseline rates of reported morbidity and hospitalisation (**Table 3**). However, latrine ownership was higher among intervention households than control households. Amongst insured women, a higher proportion had attended school and a higher proportion lived in a concrete home in the intervention arm. The between cluster coefficients of variation (*k*), estimated using baseline hospitalisation data, were 0.49 (urban) and 0.56 (rural). At baseline, the three focus conditions – fever/malaria, diarrheal illness and hysterectomy – comprised approximately half of all hospitalisations in the preceding 6 months amongst both insured and uninsured women (48 of 99 hospitalisations). Hysterectomy was the most common reason for hospitalisation. Hospitalisation rates among insured women were approximately double those among the uninsured.

**Table 3:** Baseline demographic characteristics, by insurance status and treatment arm

	Uninsured (n = 980)		Insured (n = 954)	
**Selected variables**	**Intervention (n = 490)**	**Comparison (n = 490)**	**Intervention (n = 4698)**	**Comparison (n = 485)**
Mean age in years	37.0	35.9	39.8	39.1
Mean household size	5.8	5.8	6.0	5.8
% concrete home	26.1	24.9	35.1	24.1
% with toilet	60.0	51.8	63.1	46.1
% individual drinking tap	76.7	75.5	76.7	73.3
Mean annual income (INR)	82 707	80 812	82 747	76 637
% never attended school	50.2	53.9	950.1	62.7
% respondents reported illness, past 30 d	13.5	12.0	15.9	19.2
% respondents reported hospitalization, past 6 mo	3.1	2.9	7.0	7.7

INR – Indian rupee

### Intervention coverage

In the end line survey, 30.3% of insured women and 18.2% of uninsured women in intervention clusters reported attending at least one session on diarrhoea, malaria or hysterectomy in the past year (**Table 4**). A lower proportion of women reported attending hysterectomy sessions compared to diarrhoea and malaria. Of 203 surveyed women who reported participating in a session, women who were insured, currently working and had attended at least primary school were more likely to attend.

**Table 4:** Intervention outreach by insurance status (% women surveyed intervention areas, n = 833)

	Malaria	Diarrhoea	Hysterectomy	Any session
Insured	23.2	25.0	13.2	30.3
Uninsured	13.6	14.1	6.3	18.2

### Intervention effect on claims, hospitalisation and morbidity

During the 18–month intervention period, 3340 women residents in the study area were insured at some point, contributing 1436 person–years in the intervention arm and 1227 person–years in the comparison arm. These women submitted 140 claims for the three target conditions over the study period, with a slightly higher claims rate (5.5 per 100–person years) in the intervention arm, compared to 5.0 in comparison clusters. The estimated rate ratio, adjusted for location and cluster–level baseline claims rate was 1.03 (95% CI: 0.81–1.30, *P* = 0.81) (**Table 5**). There was no evidence that the effect of the intervention differed between rural and urban areas (*P* = 0.84).

**Table 5:** Estimates of the effect of the intervention on claims, hospitalization and morbidity rates for three focus conditions using individual–level data, Poisson regression random effects model

	Intervention (14 clusters)	Comparison (14 clusters)	Effect estimate	95% CI	*P*
Effect on claims*					
Claims for diarrhoea, fever, hysterectomy	79	61			
Total person–years	1756	1227			
Claims rate/100 person–years	5.50	5.04	1.03	0.81, 1.30	0.81
**Effect on hospitalization rates†**					
Total episodes of 3 conditions	36	31			
Total person–years	1355	1279			
Hospitalization rate/100 person–years	2.66	2.42	1.05	0.58, 1.90	0.88
**Effect on morbidity rates†**					
Total morbidity episodes, 3 conditions	157	140			
Total person–months	2705	2606			
Morbidity rate/100 person–months	5.80	5.37	1.09	0.87, 1.38	0.46

CI – confidence interval

*Adjusted for urban/rural location and cluster–level baseline claims rate.

†Adjusted for insurance status, location, survey round and cluster level baseline rate.

**Figure 3:**
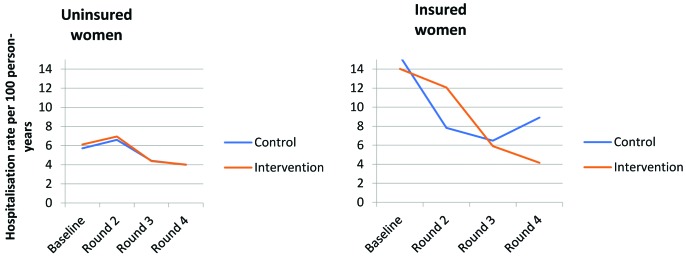
Hospitalisation rates by survey round, using insurance status at baseline.

The post–intervention hospitalisation rate for the three conditions was 2.7/100 person–years in the intervention arm, compared to 2.4/100 person–years in the comparison arm. After adjusting for insurance status, rural/urban location, survey round and baseline hospitalisation rates, there was no evidence of an intervention effect on hospitalisation rates for the three conditions (**Table 5**). There was no evidence of effect modification by insurance status (*P* = 0.91) or by rural/urban location (*P* = 0.18). Adjusting for imbalanced demographic characteristics identified at baseline and other potential covariates did not result in an important change in the point estimate or improve statistical efficiency (data not shown). Among initially insured women, hospitalisation rates decreased by approximately half in both intervention and control areas compared with pre–intervention rates, with smaller decreases observed among uninsured women (**Figure 3**).

Fever/malaria, diarrheal illness and gynaecological conditions comprised between 35–56% of reported morbidity in the past 30 days at baseline, with very few instances of gynaecological morbidity reported. The between cluster variation coefficients (*k*), calculated using baseline data, were 0.40 (urban) and 0.19 (rural). The post–intervention morbidity rate in the intervention area was 5.8/100 person–years, compared to 5.4/100 person–years in the control arm. There was no evidence of an intervention effect on morbidity for the three conditions (**Table 5**) or evidence of effect modification by insurance status (*P* = 0.75) or rural/urban location (*P* = 0.37).

### Process findings

Observations of 20 education sessions noted two main findings regarding implementation quality: (i) a uniform, structured message was provided and reinforced by print media by most CHWs and (ii) CHW communication abilities varied considerably. The majority (83%) of participants interviewed within a month after attending an education session reported knowing how to control mosquitoes, while 45% reported knowing that handwashing with soap is an effective measure to prevent diarrhoea. Neither quantitative survey data nor qualitative interviews provided evidence of changes in behaviour related to handwashing or mosquito prevention, however. Health workers pointed to underlying determinants such as poor sanitation and lack of quality outpatient treatment as persistent barriers.

**Figure 4:**
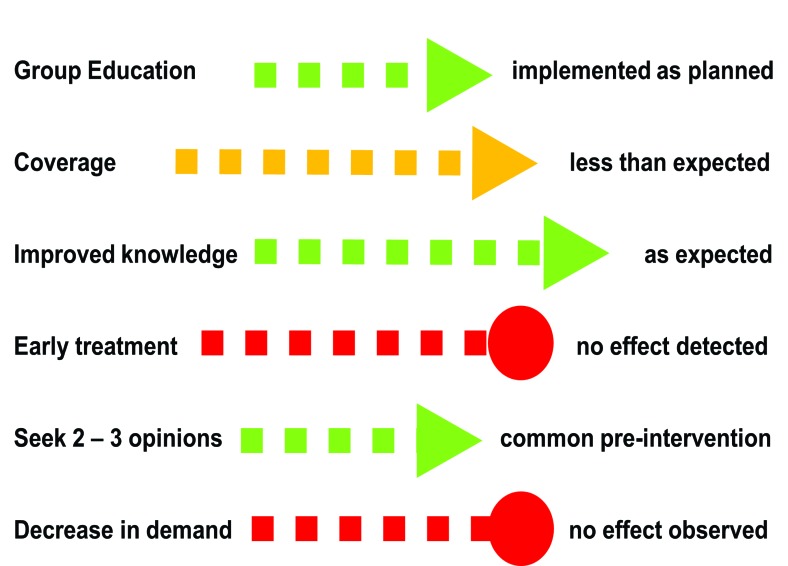
Summary of process findings for hysterectomy. Steps in causal pathway as defined in **Figure 2**.

Regarding hysterectomy, 90% of women reported they would normally seek at least two opinions before undergoing hysterectomy. In–depth interviews with 10 participants suggested they gained: (i) increased knowledge and (ii) confidence in the local CHW as a resource person. However, understanding of potential side effects and the risk of premature menopause was low. Further, most women interviewed expressed that, despite learning new information, doctors’ opinions would be the most important factor in a treatment decision. Interviews with CHWs and participants did not suggest a reduction in women’s demand for hysterectomy, to the extent that such interviews could assess women’s attitudes (**Figure 4**).

## DISCUSSION

In this setting, a high proportion of hospitalisations in adult women for diarrhoea, fever and hysterectomy was confirmed through both insurance claims and a household survey – indicating that the intervention targeted the conditions responsible for a major component of treatment sought by adult women in the study population. Although process findings suggested improvements in knowledge, we found no evidence of an effect of the intervention on care–seeking or health outcomes. These findings depart from several published studies that have reported evidence of an effect of CHW–led group health education on preventive health behaviours and some health outcomes related to malaria, family planning and women’s health [9–16]. There are few published randomised trials, however, that do not report a positive effect of CHW–led group education on treatment–seeking or health outcomes, which may reflect both publication bias and, possibly, limited use of CHW–led group education as a strategy.

Three trials conducted in the United States of CHW–led group education sessions to improve screening rates for cervical and breast cancer amongst women in ethnic minority communities reported improved uptake of screening tests, two of which indicated that behaviour change was associated with improved knowledge. The third trial indicated that while group education improved breast cancer screening, it was more likely CHWs’ social position and influence amongst women, rather than improved knowledge among the participants, that mediated behaviour change [15]. Non–randomised evaluations in India have reported increased health knowledge amongst women exposed to group education, with some evidence pointing to changes in treatment–seeking behaviour [25–27].

The trial evaluated an add–on education intervention in an ongoing CHW program that could have been easily scaled–up if found effective. The intervention delivery mechanism was aligned with two established characteristics of effective CHW programs: SEWA CHWs were embedded in their communities and were supported through continuous training and management inputs [5,28]. Intervention coverage was low, however. The intervention only included three sessions per month per CHW, because SEWA CHWs were already fully tasked with existing responsibilities and potentially overburdened – a common challenge to CHW programs that prevents greater coverage of interventions [5]. Nonetheless, CHW–led group education efforts in other settings suggested that even low numbers of meetings can trigger changes in behaviour [14,15]. In these cases, however, the intervention outcome was receipt of either a pap test or mammography– both one–time, preventive actions with logistical support by a CHW and readily available health services.

### Barriers from knowledge to behaviour change

Interventions that have demonstrated evidence of an effect on handwashing were considerably more intensive campaigns that included distribution of soap. A handwashing campaign in Karachi, Pakistan that included weekly education as well as soap distribution reported a sustained effect on handwashing with soap, while a rural Indian education–only intervention did not detect evidence of improved behaviours [29–31]. Accordingly, SEWA’s education sessions may not have been sufficiently intensive or targeted to trigger a change in preventive behaviour.

Regarding hysterectomy, qualitative research suggested that women who had undergone hysterectomy had previously suffered from untreated gynaecological morbidity such as excessive menstrual bleeding or fibroids that disturbed daily life and work [32]. Primary gynaecological care was not available, and providers typically suggested hysterectomy as a first– or second–line option for women who had completed childbearing. Lack of knowledge of side effects and sociocultural attitudes towards women’s reproductive systems led providers and women to believe that the uterus was not a required organ once childbearing was complete. Most women were daily wage workers without any social protection; they explained that they chose to undergo the procedure to preserve their health and productivity. Although the intervention appeared to improve knowledge of hysterectomy and its side effects, it did not address these underlying health systems or sociocultural determinants.

### Evaluation

This trial is that it was powered to detect a large (40%) reduction in claims for the three focus conditions. Though the data do not suggest any effect of the intervention on claims rates, the wide confidence intervals around the point estimates do not preclude the possibility of a smaller effect (<30%). VimoSEWA management had indicated that a reduction lower than 30–40% would not have significant financial bearing on the claims ratio, and would not be enough to justify funding an education intervention. The baseline survey reported a smaller number of hospitalisation events per cluster (and larger k) than initially assumed, suggesting that detecting this level of reduction was likely unrealistic. Lastly, 8% of women in control clusters also reported attending education sessions on the three conditions, which could reflect recall error or contamination – a further possible reason why the evaluation did not detect an intervention effect.

Given the observed coverage of the intervention – 30% of insured and 18% uninsured women in intervention areas reported attending sessions – the evaluation was not powered to detect the level of reduction in hysterectomy, diarrhoea and fever which might reasonably be expected. To illustrate in the case of hysterectomy, the most common reason for hospitalisation: assuming that the intervention effect was limited to the 10.5% of women who reported attending a hysterectomy session and that the intervention, if effective, would not have prevented more than 50% of hysterectomies among those women, there would have been, at most, a 5% reduction in an annual incidence of 21/1000 woman years (estimated from survey data) [33] – which corresponds to approximately three cases over the study period.

### Strengths and limitations

The intervention outcomes – reported morbidity and hospitalisation rates and claims rates – were similar across arms at baseline for both insured and uninsured households, suggesting that randomisation achieved, in large part, its intended goal. We utilised claims data from the entire insured population, rather than a sample. In the survey, tracking both insured and uninsured women allowed the intervention to be examined from a community perspective, rather than solely for the insurance program. The claims data were not compromised by survey fatigue, attrition, recall errors or other limitations of self–reporting morbidity and hospitalisation that may have affected the household survey [34–39]. Lastly, the use of process and mixed methods data in addition to our randomised trial allowed us to examine its context, intended mechanisms and implementation gaps [28].

However, the decrease in self–reported hospitalisation in insured women not observed in the claims database suggests survey fatigue amongst respondents. The evaluation may have been improved by better accounting for attrition in the household survey. The initial estimate of 0.28 for k, considerably lower than that retrospectively calculated with baseline data, was based on aggregated rural and urban claims data, rather than manual categorisation into the three conditions as conducted during the trial analysis. Although the number of clusters was limited by the availability of CHWs, better estimation of between cluster–variation during the design phase would have made a stronger case to consider a larger sample size per cluster to improve power and precision, while noting the diminishing returns of increasing sample size given large values of *k*.

Regarding the intervention, our CHW–led education program may have been too ambitious in its intent to address three distinct ailments amongst both insured and uninsured women. Focusing on one condition may have allowed for wider coverage, stronger interventions and better implementation monitoring to alleviate concerns related to quality as well as contamination, although a larger number of clusters would have been required to evaluate the effect. Further, the use of formative research prior to the design might have provided important inputs on drivers of women’s behaviour. For example, the hysterectomy component might have been strengthened by inclusion of an approach to provider behaviour or social norms.

### Implications for CHW–led health education

Gaps in preventive knowledge suggest that health education remains a necessary, albeit not sufficient, intervention in this setting. Our findings also suggest that CHWs embedded in an on–going program may not be the most effective medium to disseminate information, in light of time constraints and variation in communication skills. Mobile technology could potentially standardise and support CHW–led health education efforts [40]. Mass media interventions are one alternative that does not depend on CHWs. Evaluations have reported moderate evidence for an effect of mass media on health behaviours when situated within multifaceted interventions [41]. Similarly, there is a moderate body of evidence that supports the effect of home visits by CHWs as a tool to change behaviour [3,42]. Although CHWs’ existing responsibilities and SEWA’s limited resources prevented a more intensive intervention, more structured individual follow–up and home visits could have potentially been included.

While women expressed trust in CHWs as sources of information and support in seeking treatment, our findings also suggest the need to reconsider a focus on individual behaviour change as the main goal of CHW–led health education. Earlier research conducted at SEWA suggested that women are hospitalised for diarrhoea and fever after outpatient treatment repeatedly failed [43], while hysterectomy emerged as a symptom of weak health services and embedded social norms. Thus, even with improved coverage and quality of a health education intervention, an approach premised on changing individual knowledge and action alone may have been insufficient to affect health outcomes. Instead, CHW–led interventions could have utilised group education processes to instigate collective action for improved water and sanitation, for example. Similarly, an advocacy component led by CHWs to address the drivers of hysterectomy, such as the lack of gynaecological care in primary health care settings, could have been explored. CHWs’ position as bearers of both technical knowledge and indigenous experience could have been better capitalised upon, through their roles as educator–advocates in the community.

## CONCLUSION

The high proportion of insurance claims utilised for seemingly preventable illnesses emphasises the need for design and evaluation of scalable, community–based strategies to address common causes of hospitalisation amongst low–income women. The wide coverage and reach of CHWs in India, particularly government ASHAs, is a potential opportunity to reach women with preventive health interventions. Our evaluation suggests that, while CHW–led health education was not sufficient to reduce hospitalisation, strategies that capitalise upon CHW strengths – their position in the community, practical skills and local knowledge – should continue to be experimented with for their potential to strengthen health systems and improve women’s health outcomes.
